# The Hardness and Strength Properties of WC-Co Composites

**DOI:** 10.3390/ma4071287

**Published:** 2011-07-14

**Authors:** Ronald W. Armstrong

**Affiliations:** Center for Energetic Concepts Development, Department of Mechanical Engineering, University of Maryland, College Park, MD 20742, USA; E-Mail: rona@umd.edu; Tel./Fax: +410-723-4616

**Keywords:** hardness, cracking, indentation fracture mechanics, dislocations, fracture stress, stress intensity, WC-Co composite, cemented carbide, WC, MgO, Al_2_O_3_, toughness, Hall-Petch relation, contiguity

## Abstract

The industrially-important WC-Co composite materials provide a useful, albeit complicated materials system for understanding the combined influences on hardness and strength properties of the constituent WC particle strengths, the particle sizes, their contiguities, and of Co binder hardness and mean free paths, and in total, the volume fraction of constituents. A connection is made here between the composite material properties, especially including the material fracture toughness, and the several materials-type considerations of: (1) related hardness stress-strain behaviors; (2) dislocation (viscoplastic) thermal activation characterizations; (3) Hall-Petch type reciprocal square root of particle or grain size dependencies; and (4) indentation and conventional fracture mechanics results. Related behaviors of MgO and Al_2_O_3_ crystal and polycrystal materials are also described for the purpose of making comparisons.

## 1. Introduction

Previously, Armstrong and Cazacu [1] have presented an analysis of the hardness and indentation fracture mechanics (IFM) properties reported for a number of WC-Co materials and as related to comparative measurements made on alumina materials. Use was made of a continuum mechanics description of cracking with an associated plastic zone at the crack tip and, which analysis when combined with a Hall-Petch (H-P) description of an inverse square root of grain size dependence for the crack-free fracture stress, provided a relationship for the combined effects of crack size, grain size and plastic zone size on measurements of the fracture mechanics stress intensity, K_C_:

K_C_ = σ_K_[πc]^1/2^ = α'_K_s^1/2^[σ_0C_ + k_C_ℓ^−1/2^] (1)

In Equation (1), σ_K_ is the applied stress, c is an appropriate pre-crack size, α'_K_ is a numerical factor that is equal to (8/3π) for the mode I, plane strain, fracture mechanics stress intensity, K_1c_; s is the plastic zone size at the crack tip; σ_0C_ is the friction stress for dislocation movement within a crack-initiating dislocation pile-up; k_C_ is the corresponding pile-up stress intensity; and, ℓ is the effective material grain size [2].

In the present article, the hardness and strength properties of the WC-Co system are further reviewed with regards to the background plasticity and cracking measurements leading to the application of Equation (1) to the fracture mechanics properties of a number of WC-Co materials. Elastic, plastic and cracking measurements are presented on a hardness stress-strain basis for alumina crystals and polycrystals and, especially, these same type measurements made on MgO crystals are also described with regard to the thermally-activated viscoplastic properties of dislocations and their role in crack initiations. In this regard, MgO, which is an important “ductile ceramic” whose dislocation mechanics based deformation and cracking behaviors have been extensively investigated, provides a useful connecting link with the somewhat lesser known deformation and cracking properties of Al_2_O_3_ crystals and even less so of WC crystals; see, for example, the review article on ceramic crystal deformations in *Dislocations in Solids* [3].

## 2. Hardness Stress-Strain Measurements

**Figure 1:**
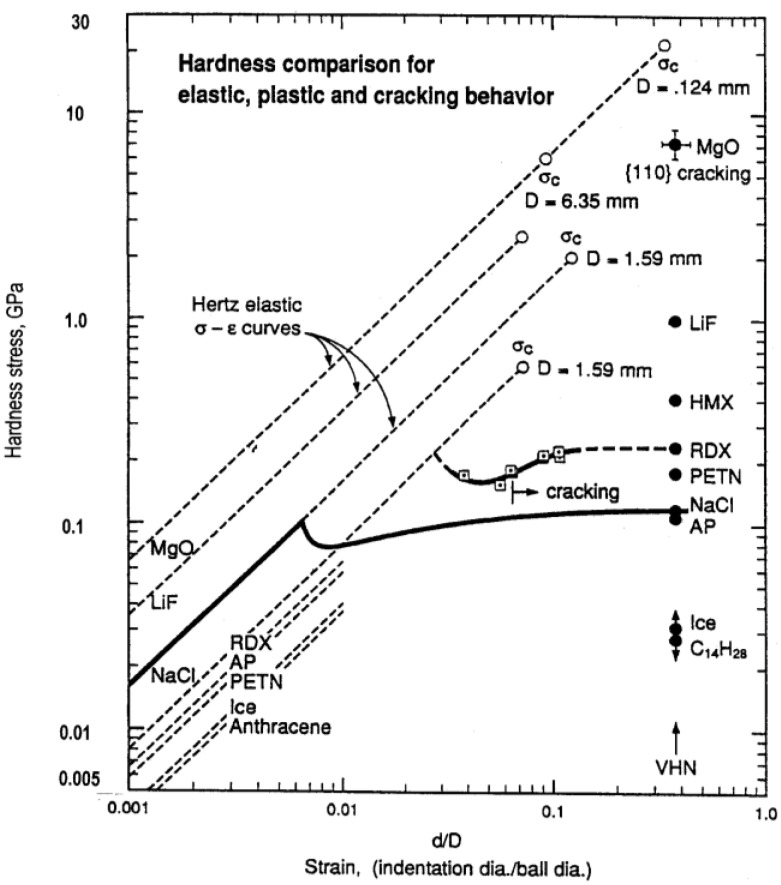
Elastic, plastic, and cracking hardnesses compared on an effective stress-strain basis for MgO and related crystals [4].

To start, Figure 1 shows a comparison of the hardness properties of MgO crystals relative to the hardness properties of a number of other ceramic-like energetic crystals and of several other generally “softer” crystals [4].

In Figure 1, the hardness stress is expressed as the mean pressure on a surface-projected contact area of diameter, d, obtained under a spherical ball indenter having diameter, D. The effective strain is expressed as (d/D). The hardness stress under load may be determined for elastic loading by measuring the penetration depth, h_e_, and determining d_e_ in accordance with the relationship d_e_ = [2h_e_D]^1/2^. For plastic straining, d = 2[h(D − h)]^1/2^, that is determined on the basis of the ball being rigid, and for which d is observed to be unchanged during unloading, at least, for d > d_e_.

The solid and dashed linear dependencies of hardness stress, σ_H_, on (d/D) are given in Figure 1 as determined from the Hertz relation

σ_H_ = (4/3π){[(1 − ν_S_^2^)/E_S_] + [(1 − ν_B_^2^)/E_B_]}^−1^(d_e_/D) = (4/3π)E_r_(de/D) = (4/3π)E_r_(2h_e_/D)^1/2^ (2)

In Equation (2), ν_S_, E_S_ and ν_B_, E_B_ are the Poisson’s ratio and Young’s modulus for the specimen and ball, respectively. In Figure 1, ν_B_ and E_B_ were taken as 0.28 and 204 GPa, respectively, for a steel ball indenter for both the dashed lines and the linear part of the continuous loading curve shown for the NaCl crystal result. The last equality in Equation (2) involving h_e_, establishes a direct determination of σ_H_ in a continuous loading curve obtained with a nanoindentation tester for which D can have been measured separately or determined by fit to the measured curve of displacement dependence on the applied load. In Figure 1, diamond pyramid hardness test results are plotted for the listed materials at a representative value of (d/D) = 0.375. At the terminal values of a number of the Hertz-type lines, various points are identified, with associated D values, for the cracking stresses, σ_C_, determined on an indentation fracture mechanics (IFM) basis from the relationship:

σ_C_ = {4E_S_γ'/[πD(1 − ν_S_^2^)(κ_1_^2^ + κ_2_^2^)]}^1/2^(d/D)^−1/2^ (3)

In Equation (3), γ' is the crack surface energy and the dimensionless factor (κ_1_^2^ + κ_2_^2^) = 2.5 × 10^−5^, as described for indentation fracture mechanics measurements made on silicon crystals [5]. For the terminal dashed line of the MgO crystal, the D = 0.124 mm value was determined from the average indentation diagonal length of the diamond pyramid indentations. For the dashed MgO line, ν_S_ was taken as 0.181 and E_S_, as 312 GPa.

The usefulness of Figure 1 is demonstrated in one case by the comparison of adjacent Hertzian lines and indentation measurements that are shown for NaCl and RDX (cyclotrimethylenetrinitramine) crystals. The comparison reveals that RDX is relatively compliant in its elastic deformation while being plastically hard and, because of its lower σ_C_ value, brittle. The respective characteristics are explained for RDX in terms of its molecular bonding, difficulty of dislocation motion, and lower surface energy for cracking. On the basis of such results, the plastic flow of crystals cannot be gauged in terms of a constant ratio of hardness and elastic modulus. Another consideration to be noted for the plotted MgO and RDX crystal results is that cracking has occurred at lower plastic hardness values than the elastic IFM value; and this occurs because of the role of dislocation pile-ups in initiation of cleavage cracking [6]. More is to be said about this in the present review. For MgO, the close proximity of the Hertzian elastic loading curve to the plastic indentation measurements enables the understanding of the exaggerated shapes shown in residual diamond pyramid indentations that, for example, are concave-shaped when the indenter sides are aligned parallel to <100> directions on an (001) crystal surface and have a convex shape for <110> aligned indenter sides [7].

## 3. Hardness Comparisons of MgO, Al2O3 and WC Crystals

An important advantage of the load and displacement sensitivities of modern nanoindentation hardness testers is that the initial material elastic deformation responses of the type indicated in Figure 1 are able to be easily measured in a continuously recorded load-deformation curve.

**Figure 2:**
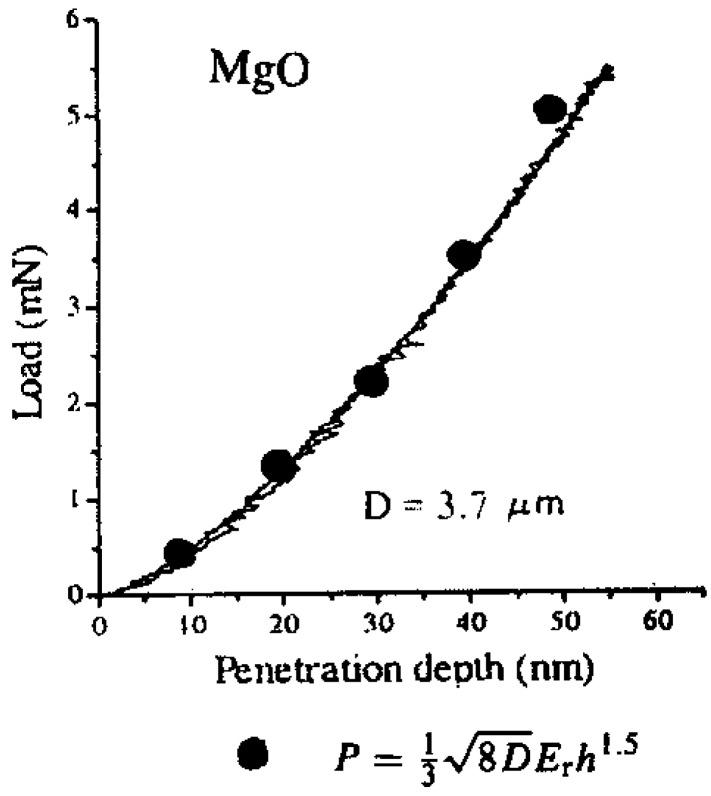
The load/unload response of an indented (001) MgO crystal surface [8] and points fitted to the listed Hertzian relation for load and penetration depth [9].

**Figure 3:**
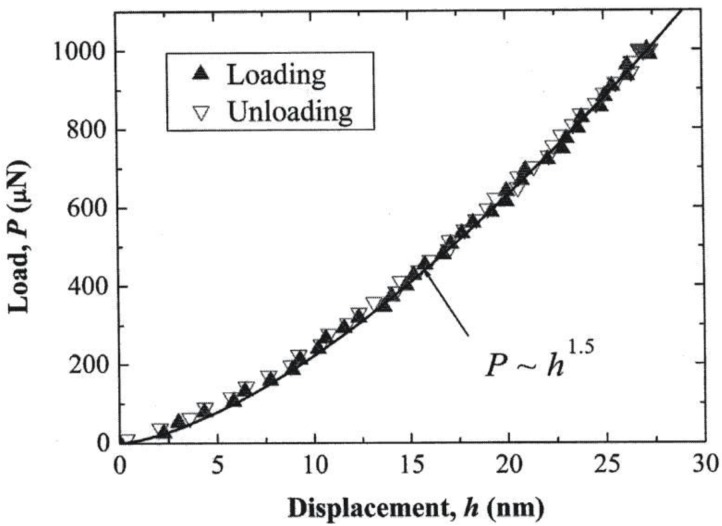
The elastic load/unload response of an indented (0001) Al_2_O_3_ crystal surface [10].

In Figure 2 and Figure 3, ν_B_ and E_B_ were taken as 0.068 and 1145 GPa for a diamond indenter. As shown in the legend within Figure 2, the MgO elastic curve was fitted with a relatively large value of D = 3700 nm. For the Al_2_O_3_ result in Figure 3, D was reported as 408 nm and ν_S_ and E_S_ were taken as 0.235 and 425 GPa, respectively. Otherwise, in any test in which D would be measured separately, as was done for macro-scale continuous indentation measurements made on lignin and solid and porous aluminum materials, estimations could be made of ν_S_ and E_S_ values.

**Figure 4:**
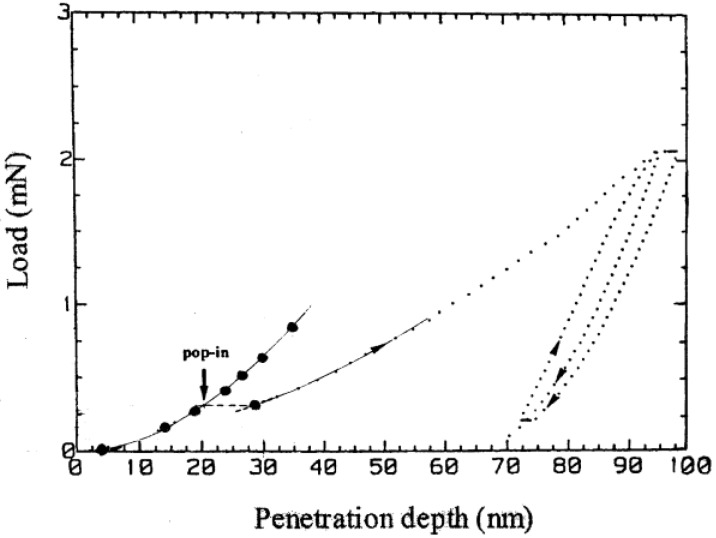
An elastic/plastic loading curve for nanoindentation of an MgO (001) crystal [13,14].

The totally elastic nanoindentation result in Figure 3 compares favorably with an earlier description of the elastic portion of a similar test result on an Al_2_O_3_ crystal that was carried into the plastic regime [9,11,12] Figure 4 shows the elastic fit applied to one such loading curve reported for another indented MgO (001) crystal surface [13,14]. In this case, D was determined to be much smaller at 400 nm compared to that determined for the separately reported elastic result in Figure 2. The consequence of the different D values on the two overall loading curve results is easily discernable in the plastic deformation and unloading results, for example, in exhibiting a much steeper unloading curve after plastic deformation for the smaller D curve [14]. Thus, by comparison of the two MgO curves shown here, a much greater (d/D) strain is achieved at the same penetration depth with a smaller D.

**Figure 5:**
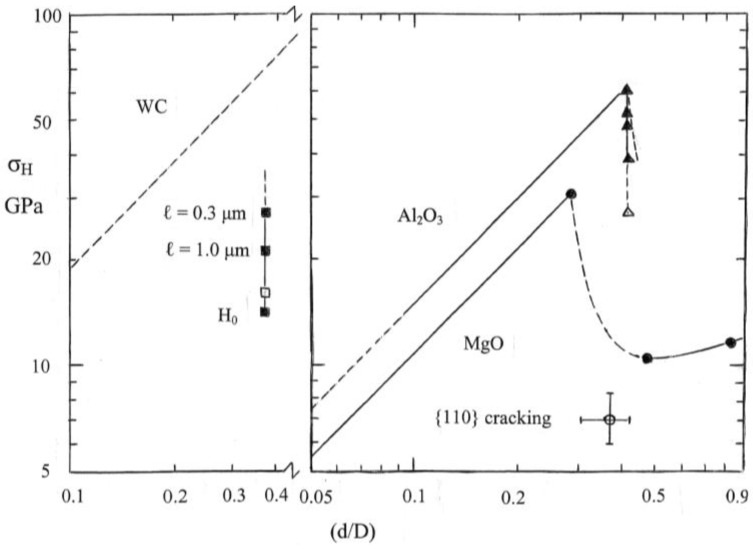
Comparative hardness stress-strain dependencies for MgO [4,13], Al_2_O_3_ [10,15] and WC [15,17] crystals; in the latter case, only reference crystal (open square) and Hall-Petch type (filled square) micro-hardness values are plotted for comparison with the dashed Hertzian line.

The characteristic “pop-in” displacement for initiation of plastic flow is well-marked in Figure 4, and, furthermore, the ensuing continuation of the deformation curve is associated with the lesser dependence of load on penetration depth during the progress of plastic deformation. On the other hand, the unloading and reloading curves illustrate the essentially elastic deformation behavior associated with the flattening or re-indentation occurring at the base of the residual plastic indentation. Figure 5 shows the effective MgO stress strain curve determined for the combined elastic and plastic behavior in Figure 4. In this case, beginning from the onset of plastic deformation signaled by the pop-in displacement, the total h measurement is taken to be given by the rigid ball result.

The linear Hertzian and subsequent plastic deformation segment shown in Figure 5 for MgO applies for the initial elastic loading curve of Figure 4. The dashed load drop segment applies for the pop-in displacement and the follow-on solid curve for increase in hardness stress with increase in deformation, now from a yielded lower hardness level, corresponds to the full extent of the loading curve measurement in Figure 4 taken to the point of unloading at (d/D) = 0.83. The open circle point with indicated limits for typical values of microhardness measurements on MgO crystals and generally involving {110} cracking is the same point shown in Figure 1. The occurrence of higher plastic hardness values for the nanoindentation test result of Figure 4 are typically characterized as an “indentation size effect”, ISE, that occurs for hardness indentations made at smaller contact dimensions. The ISE is associated with the observation that the nucleation and propagation of dislocations and cracks depend on their effective size-scales and distributions, especially at sub-micron dimensions.

More limited hardness results are shown in Figure 5 for the Al_2_O_3_ and WC hardness stress-strain results but the points that are shown have their own interesting characteristics for comparison with the MgO results. For the Al_2_O_3_ stress-strain result, the solid elastic line extending to the top triangular point, covers the recorded deformation up to the onset of pop-in deformation and was determined from the reported elastic loading measurements [10]. The lower (solid) triangular points are hardness measurements selected from a larger number of plastic hardness values reported in the same investigation. The reported measurements, obtained with a Berkovich (triangular) indenter system and plotted here at a (d/D) value of 0.42, were calculated by performing load/unload measurements at various points along the plastic deformation curve and utilizing an effective plastic area function importantly adjusted for the elastic unloading. Such results are to be compared with the open triangle point, which is a representative hardness value of 2700 kgf/mm^2^ that has been reported for the conventional diamond pyramid microhardness of Al_2_O_3_ crystals [15], and thus is in reasonable agreement with expectation of the ISE effect. An extreme ISE effect or difficulty in accounting for the true indenter tip geometry would seem to be required in order to explain *in-situ* nanoindentation hardness values of 150–170 GPa for the WC phase and 20–40 GPa for the Co binder phase in a WC-Co composite material [16].

In Figure 5, the (left-side) WC hardness results have been shifted so as to provide clarity for the dashed Hertzian line and the associated hardness points. In this case, the open square point is a representative diamond pyramid hardness value of 1630 kgf/mm^2^, also obtained from reference [15] (p. 134). The other (solid) square points have a quite different origin that relates to the main purpose of the present review. These points were determined in accordance with the following H-P type of hardness relation that was utilized to quantitatively account for the hardness properties measured for WC crystal or grain constituents in the WC-Co composite system [17]:

H = H_0_ + k_H_d^−1/2^ (4)

In the H-P model description of Equation (4), H_0_ is taken as an average hardness for a WC crystal particle over all possible orientations, k_H_ is the hardness microstructural stress intensity for breakout of a slip band stress concentration from within an average WC particle, and d is the average diameter of a particle. The value of H_0_ = 1382 kgf/mm^2^ plotted in Figure 5 from the reported H-P measurements compares favorably with the reported conventional microhardness measurement. As will be subsequently reviewed here, the higher hardness values plotted in the figure correspond to hardness determinations obtained from Equation (4) with k_H_ = 23.1 kgf/mm^2^ and indicated particle sizes of 1.0 and 0.3 µm. A comparison of the conventional hardness measurements for WC and Al_2_O_3_ shows that the ambient temperature hardness of WC is lower—but that is not the full story! As will be seen below, issues of single crystal *versus* polycrystal plasticity and a role for Hall-Petch type grain size dependencies are to be involved.

## 4. Dislocation Mechanics and Thermal Activation

**Figure 6:**
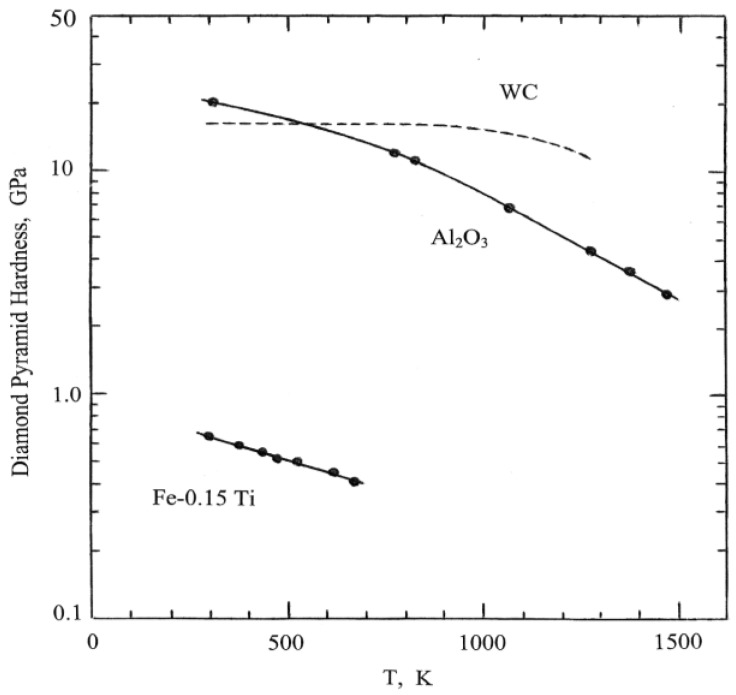
Temperature dependence of hardness for WC and Al_2_O_3_ [15] and a Fe-0.15 Ti alloy [19].

A particular advantage of WC in WC-Co composites is that the strength of the carbide particles is maintained at higher temperatures. Figure 6 shows a comparison of the temperature dependencies for polycrystalline WC and, apparently, for single crystal sapphire (Al_2_O_3_) materials as obtained from temperature dependent hardness results reported in reference [15] (p. 135 and p. 185). The results may be compared with similar measurements reported for the hardness of other WC and WC-Co materials in reference [18], in which a detailed description is given of the experimental aspects of performing the measurements. The much lower “hot hardness” of an “interstitial-free” Fe-0.15Ti alloy material is also shown in Figure 6 for comparison with a metal system [19]. Viewed at rising temperatures, the comparison demonstrates that the higher hardness of WC becomes essentially temperature independent at ~1000 K while the hardness of Al_2_O_3_ is lower than that of WC until rising above it at ~500 K. Such leveling off of either the hardness or plastic flow strength of materials at effectively lower temperatures is not unusual and is generally explained in terms of the temperature dependent part of the strength property being governed by thermally-activated dislocation motion and the leveling of strength. This is accounted for either in terms of a change in atomic coordination occurring in the cores of dislocations akin to a chemical phase change or, in other instances, by the hardness being governed by the initiation of cracking, say, as for a type of ductile-brittle transition.

Beyond the difficulty of performing hardness tests at elevated temperatures, there is the problem of interpretation of the measurements in the same manner as done for the naturally separated plastic yield and strain hardening properties of crystals or polycrystals in conventional tensile or compression tests performed as a function of temperature and strain rate. No hardness results are presented in the dislocation-based review of ceramic crystal properties given in [3]. And such temperature measurements that are shown, for example, for the critical resolved shear stresses for plastic flow of MgO crystals on both the easy {110} and more difficult {100} slip systems are at stress levels significantly lower than the conventional hardness levels, for example, respective shear stresses of ~0.02 and ~2.0 GPa are estimated for results presented at ambient temperature. A useful reference for the higher temperature deformation of MgO in compression is [20]. On the other hand, measurement of the model dislocation parameters proposed to gauge the thermally-activated mechanisms involved in the viscoplastic behavior of MgO and similar ceramic materials appear to behave similarly to those proposed for metals. A higher intrinsic friction of thermally activated flow in a ceramic crystal has been reported [3,21]. One such relevant parameter in the thermal activation model is the dislocation activation volume, v*, that, although measured in volumetric dimensions, is actually an activation area multiplied by a dislocation Burgers vector and is evaluated as

v* = kT[∂**ln**(dγ/dt)/∂τ_Th_]_T_ (5)

**Figure 7:**
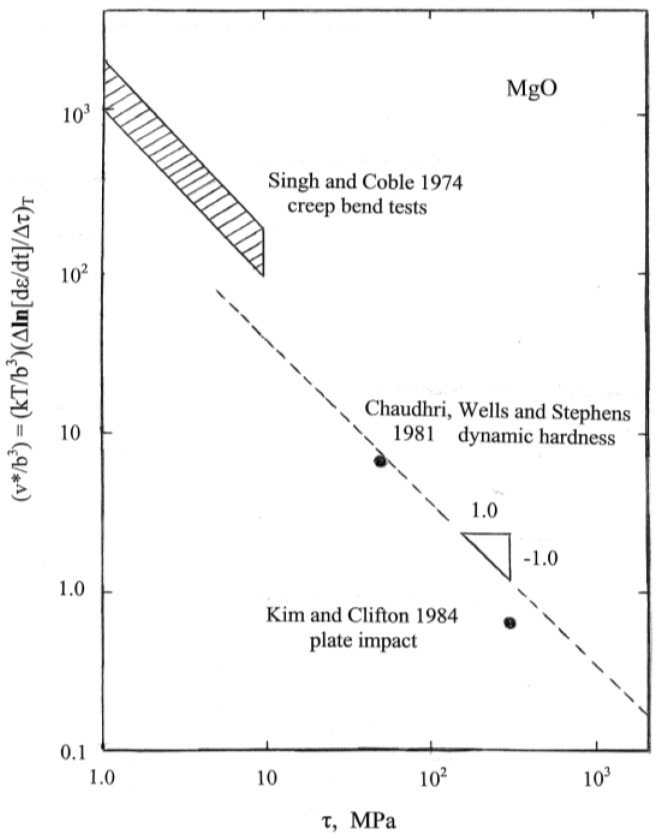
The activation volume characterization of thermally-activated dislocation motion [22,23,24].

In Equation (5), k is Boltzmann’s constant, (dγ/dt) is the plastic shear strain rate, τ_Th_ is the thermal component of the total applied shear stress, and T is absolute temperature. In many cases, v* is found to depend inversely on τ_Th_ and the consequence is that the thermal component of stress shows a logarithmic dependence on temperature as confirmed in [3]. Figure 7 shows an additional compilation for MgO crystals of reported v* measurements obtained in the listed investigations [22,23,24] spanning a range of bend test, hardness and shock plate-impact test results.

**Figure 8:**
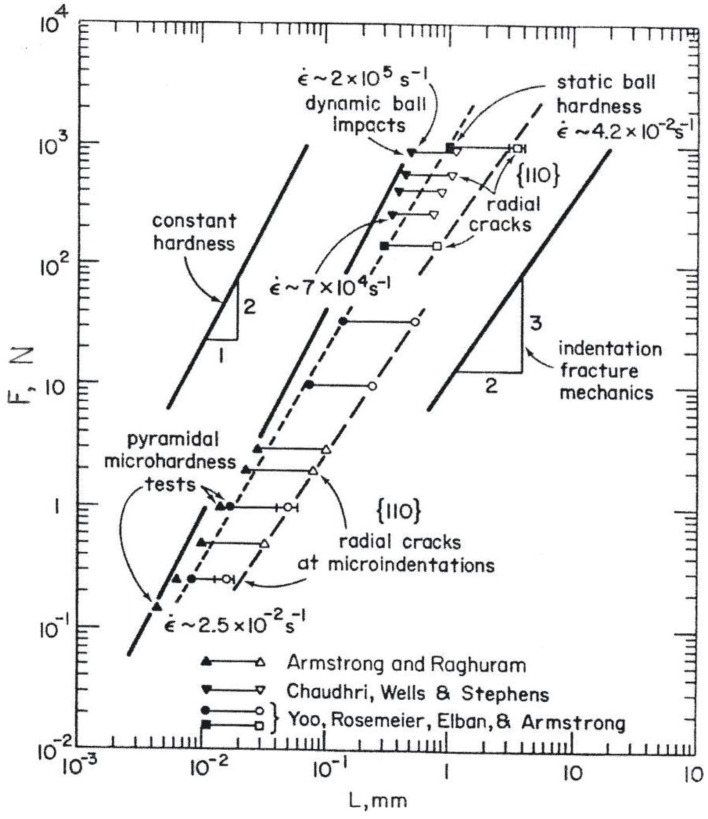
Compilation of static and dynamic hardness measurements for MgO crystals [23,26,27].

In Figure 7, the topmost cross-hatched area covers an extensive range of experimental etch pitting measurements made of dislocation velocities in MgO crystals at ambient, 373 and 423 K temperatures, all consistent with a logarithmic dependence of flow stress on temperature. The smallest value of (v*/b^3^) in Figure 7 was obtained from tabulated results presented in reference [24]. The magnitude of (v*/b^3^) = 0.65 relates to similar values reported for the shock-induced deformation properties of copper and iron materials [25]. Of greater interest is the transformed dynamic hardness point in Figure 7 that was obtained from important high rate ball impact measurements reported at strain rates of ~7 × 10^4^ and ~2 × 10^5^ s^−1^, as shown below in the compilation of MgO hardness results of Figure 8 [23,26,27]. In this case, a multiplying factor of (1/35) has been employed to obtain an effective shear stress from the dynamic hardness result, as previously estimated [15] (p.11), presumably to connect an appropriate average of the above-mentioned resolved shear stresses for easy {110} and more difficult {100} slip in MgO to the measured microhardness level of ~700 kgf/mm^2^ (~6.9 GPa) shown in Figure 1 and Figure 5.

**Figure 9:**
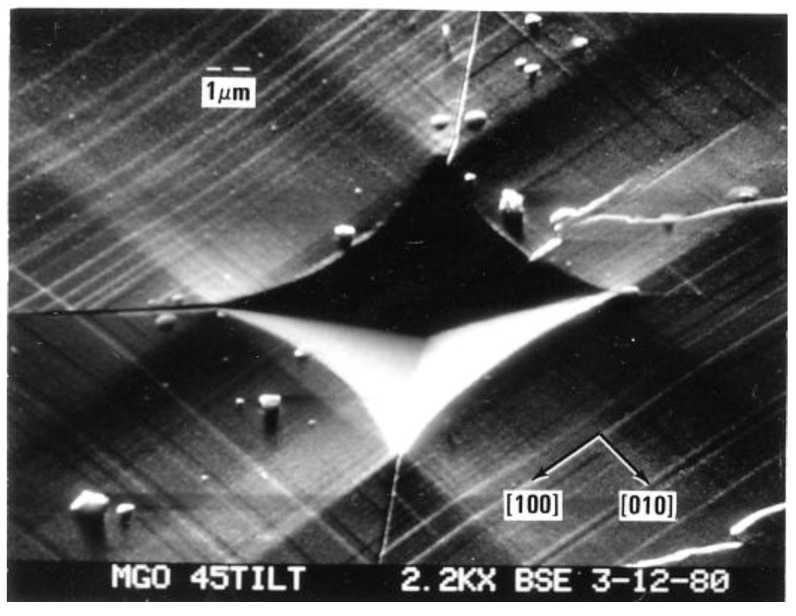
Aligned diamond pyramid indentation put into an (001) MgO crystal surface [7,28]

Also in Figure 8, the lower (triangle and circle) microhardness load values shown to have been applied to the various MgO crystals are plotted against both the residual diamond pyramid indentation diagonal lengths and the crack-tip-to-crack-tip lengths that were recorded across the indentations; the higher (inverted triangle and square) points are plotted against the residual ball diameters and associated similar crack diameters. The figure brings attention to the importance of cracking during indentation on reducing the hardness of at least certain types of crystals and, in this case to particularly reducing the hardness of MgO crystals. Figure 9, in turn, is a representative close-up scanning electron micrograph [7,28] of such cracking behavior at a [100]-aligned diamond pyramid indentation put into an MgO (001) crystal surface.

**Figure 10:**
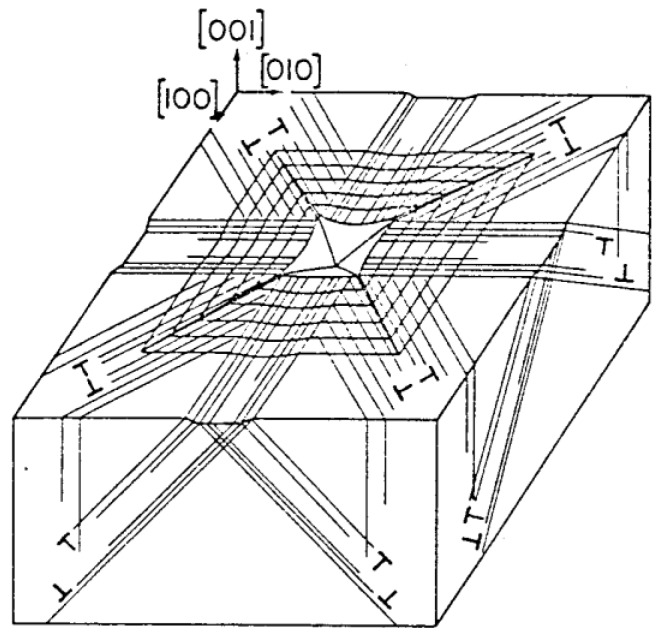
Schematic dislocation model of slip lines and {110} cracking in MgO [7].

Figure 9 and its schematic representation in Figure 10 connect, firstly, with the above-mentioned difficulty of relating hardness and shear stresses in ceramic-type crystals. Generally, in such crystals there are relatively few deformation systems to accommodate imposed deformations. Early-on in an MgO microhardness test, sessile type dislocation reactions are produced at inclined <111> type directions of volume accommodating {110} slip plane intersections and lead to dislocation pile-ups forming and producing cracks both on the (110) and (−110) planes containing the [00-1] loading direction. The stress concentrations appear to require slip on the second less-favored {111} slip systems for strain relief otherwise, as observed in Figure 9, cracking must occur across the energetically less favored {110} planes. Another important feature is that the primary indentation-forming dislocation slip spreads across the (001) crystal surface by the dislocations carrying their downward displacements in a screw orientation, so producing the “troughs” running outward from the central indentation. The latter result is uniquely tied to the crystal dislocation model for the material deformation behavior.

## 5. The Hardness of WC-Co and Al_2_O_3_ Materials

**Figure 11:**
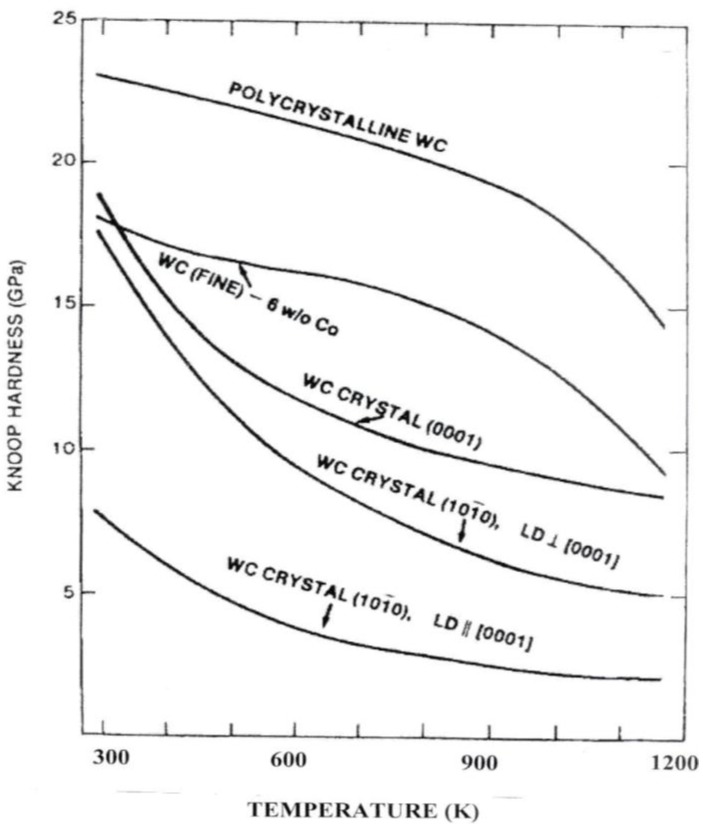
The Knoop hardness of single crystal and polycrystal WC and WC-Co alloy [18]; measurements at 9.81 N load. LD in the figure legend stands for the indenter long diagonal.

Such dislocation description as given above, especially involving dislocation pile-ups generating sufficient local internal stress concentrations so as to produce cracking is central to establishment of the Hall-Petch type dependence given for the hardness stress in Equation (4). In this regard, Figure 11 gives a comparison of temperature results in terms of the single crystal orientation-dependent Knoop hardness [18].

Figure 11, while demonstrating the usefulness of the exaggerated 7:1 Knoop indenter shape for differentiating crystallographic influences on the interpenetrating hexagonal close-packed (0001) and two directions on (10–10) surfaces of WC crystals, makes clear the distinction between single crystal hardness and the (crystal) particle size dependence of polycrystal WC and WC-Co composite material. The result for the higher polycrystal and polyphase hardnesses in the figure demonstrates that it is the H-P k_H_ that provides the added hardness of both materials over the single crystal values, at least in the temperature range shown in Figure 11. Very importantly, the result is in agreement with the description given above for the limited availability of slip systems observed for the MgO crystal hardness deformation and cracking results. The limited slip system availability is responsible for the significant H-P k_H_ value measured for WC and WC-Co materials. Such is the case too for polycrystal hcp metals [29]. Recent assessment of the ambient temperature case for polycrystalline Mg has involved estimation of a pile-up stress on the easy {0001} slip system at a shear stress of 0.3 MPa generating a local shear stress of 40 MPa for prism slip at grain boundaries [30]. Other Knoop H-P measurements have been reported for the Hall-Petch type hardness at larger grain sizes of Al_2_O_3_ materials [31]. More will be presented on this issue in connection with H-P results for Al_2_O_3_ and for the presence of a contiguity parameter, C, that provides a measure of the influence that WC-to-WC particle contacts in the WC-Co system have on hardness.

**Figure 12:**
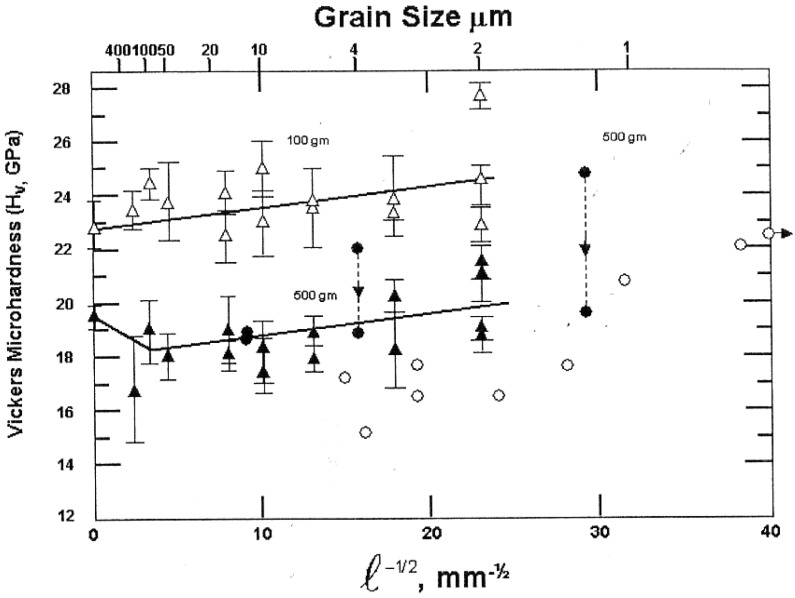
Compilation [32] of hardness measurements *vs.* grain size for A_2_O_3_ [33,34,35].

The preceding comments are not made to minimize the difficulty of reliably determining any H-P dependence in a ceramic system. For example, extensive results reported for the grain size dependence of hardness for Al_2_O_3_ materials relate to the issue as shown in Figure 12. In the figure that has been adapted from [32], the open circle points are from [33] and closed points from [34]; see also [35]. The raised level of microhardness measurements obtained at 100 g load, as compared to 500 g, are a result of the ISE; and, the scatter of measurements occurs mostly because of material porosity but at least in part because of the influence of elastic recovery on the residual indentation shapes [7,28], as mentioned also for the MgO elastic and plastic hardness strains shown in Figure 1. A further complication is a role for cracking when it occurs, as shown in Figure 9 with model description in Figure 10. A similar compilation of hardness results has been reported for other Al_2_O_3_ materials [36].

**Figure 13:**
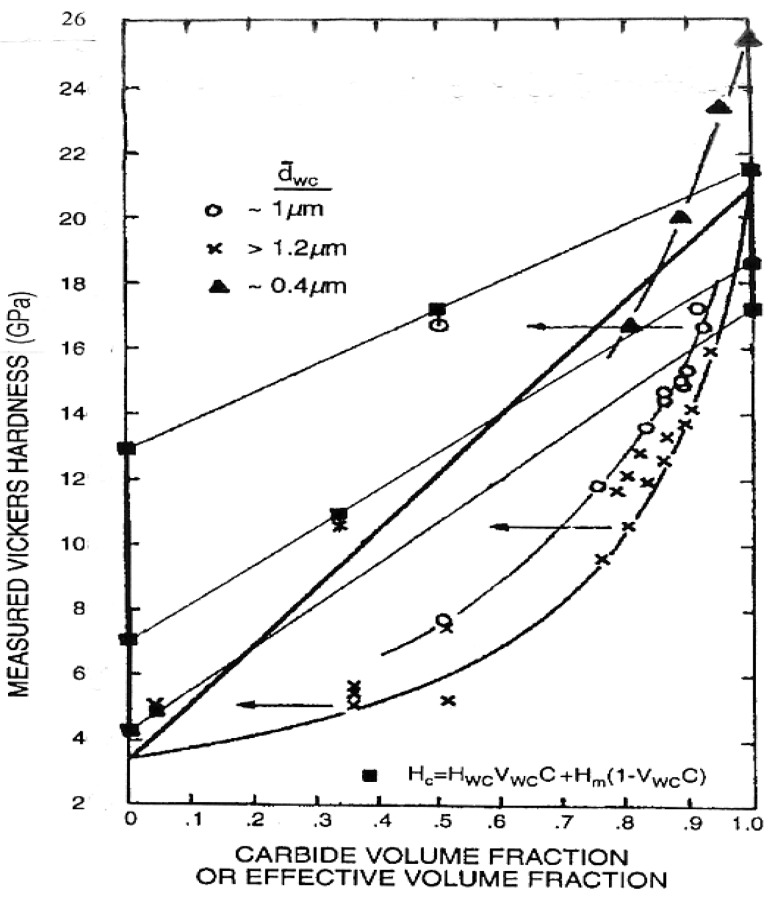
The hardness of WC-Co composites [1,17].

Figure 13 shows, first for a range of compositions of WC-Co composite materials, their hardness dependence on the volume fractions and sizes of WC particles and of mean free path length of the Co binder phases, as proposed in an important extension of the H-P method of analysis [17]. The hardness, H, is shown to follow the equation inset in the figure

H = H_WC_V_WC_C + H_m_(1 − V_WC_C) (6)

In Equation (6), H_WC_ is the H-P determined hardness of the WC particles of size d, V_WC_ is their volume fraction, H_m_ is the H-P hardness of the Co binder, and C is the contiguity measure of WC particle-to-particle contacts. Previously, the upper and lower limiting rule-of-mixtures relationships were shown to envelop the elastic modulus values, E, for the WC-Co system very well [37]. A similar approach to the lower limit relationship for hardness was modified in part because of the experimental observation of higher open circle points being obtained for the smaller WC particle sizes as compared with the lower crossed points for larger particles. To take into account such particle size influence, the rule of mixtures was modified to include particle-to-particle contacts through the effective volume fraction of particles, V_WC_C. The horizontal arrows show that the three particle positions move into such linear relationships based on the effective volume fraction and with the H-P stresses plotted in each case on the respective ordinate axes for the constituent particle and binder hardness values.

**Figure 14:**
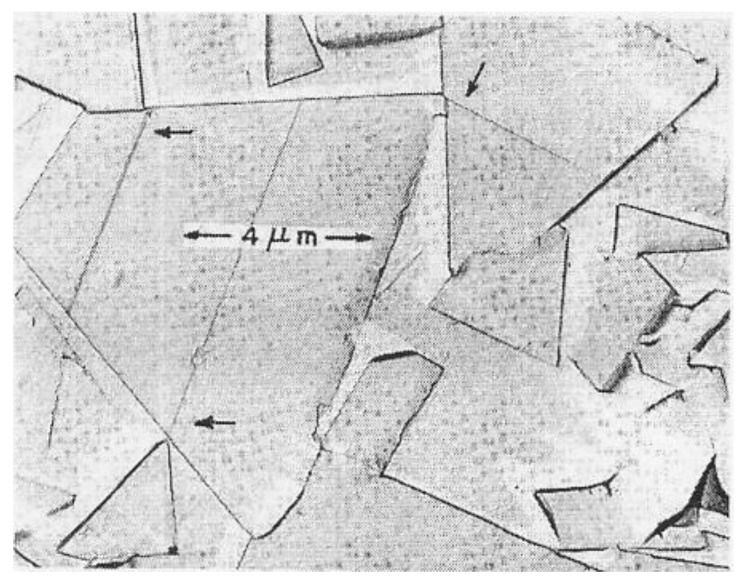
Deformation in WC-Co at WC particle-to-particle contacts [17].

The material contiguity parameter is evaluated on a stereological basis as C = [1 − (N_Co_/N_WC_)] in which N_Co_ and N_WC_ are the number of Co free paths and WC particles intersected along a suitable total line length drawn on a microstructural section [38]. An analytic derivation has been presented for C based on tabulated results from a number of investigations of the WC-Co system and with employment of a variation coefficient description applied to the WC particle size distributions [39]. Other results are reported for a theoretical model of crystallographic shapes of WC particles in WC-Co [40] and for modification [41] for flatter shapes of WC particles in WC-Ni of the contiguity-incorporated rule of mixtures relationship given in Equation (6) and Figure 13 for WC-Co. Moreover, Figure 14 shows the second important reason to consider taking the contiguity of WC particles into account in determining the composite material hardness [17]. It was pointed out at the two marked arrow positions on the trapezoidally-shaped WC particle and at the one position on the triangularly-shaped particle that slip had been initiated at the contacts between particles. Such particle-to-particle interactions are taken into account in Equation (6) so as to lessen the strengthening effect of the WC particles in the mixture and to increase the strengthening contribution from the Co binder phase while continuing to maintain a significant H-P strengthening effect on the composite from reduced WC particle sizes.

**Figure 15:**
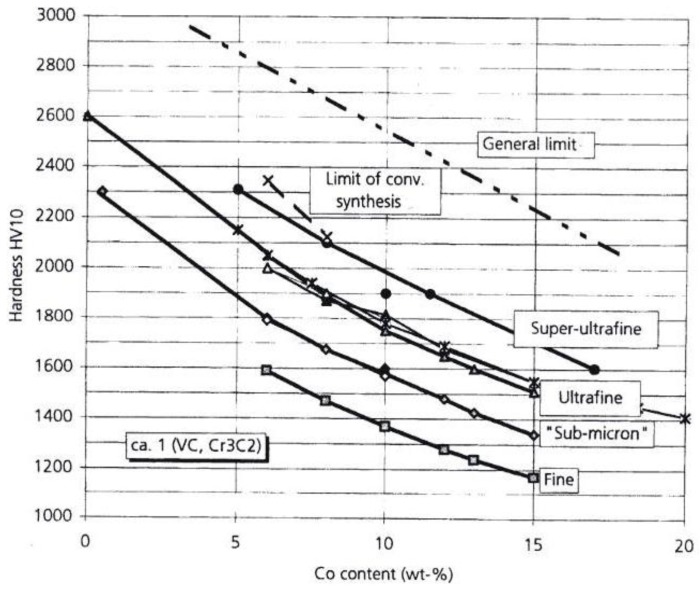
The hardness of WC-Co materials at smaller WC particle sizes [42].

The strengthening effect of smaller WC particles is further established in Figure 13 by the positions of the filled triangle points that were taken from a follow-on investigation of the influence of ultrafine or sub-micrometer WC particle sizes on the composite properties [42]. These points were taken as representative ones along the middle “ultrafine” curve shown below in Figure 15 and were plotted at these particular volume percentages of 2.5, 6 and 11 weight percentages of Co along the reasonably narrowly defined particle sizes utilized for the curve; see also Table 1 in [42]. The lower hardness results were obtained for pressed and sintered superfine and super ultrafine ingredients. Upper limiting estimations of hardness shown as the dashed line in the figure and a limit on achievable hardness to be obtained by conventional methods were plotted. The particular hardness of 25 GPa shown for 0.4 µm WC particles on the WC ordinate scale in Figure 13 may be compared with the 26.6 GPa value for 0.3 µm particles that is plotted in Figure 5 on the hardness stress-strain graph for WC. The combination of the high H_0_ and fairly low k_H_ has not produced a particularly large difference in hardness for the two particle sizes. Nevertheless, the results in Figure 15 were obtained in consideration of the possible benefit(s) to be gained in WC-Co materials composed of nano-scale constituents and also to provide an assessment of the finer WC particle sizes on the fracture toughness of the materials. Another report [43] has indicated a lesser strengthening effect at nanometer grain sizes and even weakening at the smallest nanometer dimensions. As indicated for the model description of Equation (1) for the fracture mechanics stress intensity K_C,_ there is additional influence of the plastic zone size as well as the material grain size to be taken into account for the material toughness. For the material results covered in Figure 15, K_C_ was found to decrease with decrease in particle size from a highest value of ~23 MPa.m^1/2^ (727 MPa.mm^1/2^) to a lower limiting value of ~5 MPa.m^1/2^ (~158 MPa.mm^1/2^) that was obtained for a substantial range of WC particle sizes from 3.2 to 0.4 µm. K_C_ is, even at its lowest values, generally much greater than k_H_, which, if associated with cracking, can be about three or more times larger than k_C_ in Equation (1). In other words, K_C_ >> k_H_ mainly because of the normally large value of the plastic zone size, s, and the crack-free fracture stress, σ_C_, in Equation (1) [2].

## 6. The Fracture Toughness Properties of Al_2_O_3_ and WC

**Figure 16:**
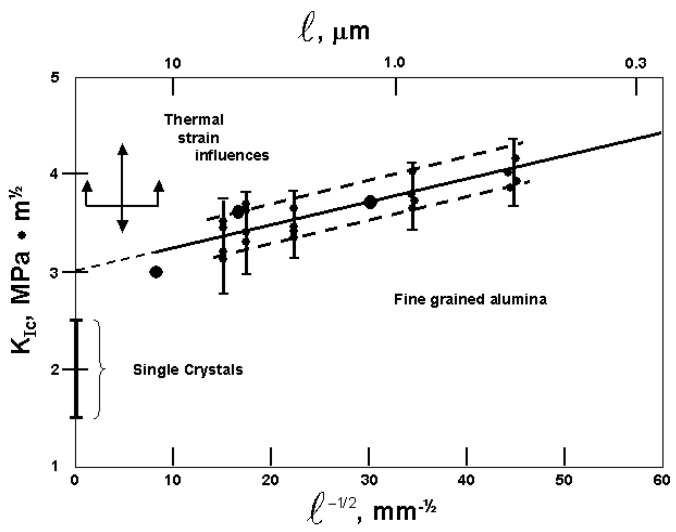
A compilation [32] of indentation fracture mechanics K_Ic_ measurements made on Al_2_O_3_ materials [48,49,50] plotted on the basis of Equation (1) with the plastic zone size, s, having a constant value.

The afore mentioned complexity of mechanical property evaluations in the WC-Co and related composite material systems has prompted alternative approaches to the evaluation of the material strength and toughness properties [44], for example, involving a fractal mechanics approach for the widest hierarchical cases of geometry of the phases and structural nature of the materials [45]. Even for the hardness properties of the WC-Co system, there is sufficient complexity to have promoted a semi-empirical approach to deciphering the influences of the WC grain size and Co mean free paths [46]. To add a further challenge of complication, there are results reported [47] for a hybrid “double cemented” WC-Co system composed of pre-fabricated WC-Co alloy granules embedded in a separately added Co matrix. Here, we proceed with a step-by-step approach to the topic by beginning with a single material case and proceeding onward to greater complication. In that regard, Figure 16 shows a compilation of results [32] for the toughness of Al_2_O_3_ materials determined from indentation fracture mechanics measurements [48,49,50].

All of the fracture toughness measurements shown in Figure 16 are lower than the lowest values reported above for the WC-Co materials. In the Al_2_O_3_ figure, the larger filled-circle points at three grain sizes were determined for Hertzian type ring cracks formed in Al_2_O_3_ substrates under loads of 30–50 kgf applied to sapphire spheres of 5 mm diameter [48]. Ring crack depths of 2 to 13 µm were associated with surface crack diameters of ~300 to ~400 µm. The same trend was followed for somewhat lower K_Ic_ measurements evaluated for the different geometry of radial cracks of ~10 µm length at diamond pyramid indentations made at the smallest applied loads of 0.5 kgf. The smaller filled-circle points shown in the figure for five grain sizes, also with the noted experimental variations and bracketed by the band of dashed lines, are average diamond pyramid determined K_Ic_ values obtained at loads of 0.3, 0.5, 1.0 and 2.0 kgf [49]. The band of measurements shown in Figure 16 are in agreement with the linear H-P type prediction from Equation (1) based on a constant value of plastic zone size. Also shown on the ordinate axis of Figure 16 is a range of K_Ic_ measurements reported for tests on Al_2_O_3_ single crystals [50]. The vertical arrows shown between the single crystal and smaller grain size polycrystal measurements mark a region where thermal strains play a role in determining the material toughness, more often than not producing higher measurements because of induced plasticity.

For larger crack sizes induced over a range of increasing applied loads from 1.0 to 25 kgf past the 0.5 kgf value utilized to obtain the filled-circle points in Figure 16, the grain size dependence of K_Ic_ was found to gradually reverse itself and, at the highest applied load of 25 kgf, to clearly decrease with decreasing grain size [48]. Table 1 shows a comparison of the diamond pyramid measurements made for the three grain sizes with application of the limiting applied loads of 0.5 and 25 kgf [1,48]. In the Table, the crack-free fracture stresses, σ_F0_ = [σ_0C_ + k_C_ℓ^−1/2^] from Equation (1), were determined from a previously reported H-P dependence [51]. The calculations for the plastic zone size, s, and for the pre-crack fracture stresses, σ_Fc_, employing the two limiting equations that are given at the foot of the Table, were obtained from a model dislocation description of critical crack growth with an associated plastic zone at the crack tip [2,52]. The larger crack size influences on the determination of a K_Ic_ that decreases with decrease in grain size is matched with reduction of the H-P dependence for σ_Fc_ but not so far as producing a similar reversed dependence. A later investigation [53] of strength, toughness and reliability of Al_2_O_3_ crack growth properties also showed greater toughness at the smallest crack sizes too but was soon reversed in agreement with those results described in Table 1.

**Table 1:** Modeled pre-crack fracture stresses [1].

ℓ, mm	K_Ic_, MPa.m^1/2^	c, mm	σ_FC_, MPa	s, mm	σ_FC_, MPa
0.0012	3.16	0.0213	536	0.01365*	335**
0.0038	3.09	0.0225	461	0.01764	305.5
0.01984	2.95	0.0245	415	0.01984	277.6
0.0012	2.93	0.3301	536	0.01735*	110.5^
0.0038	3.06	0.3239	461	0.01730	95.9
0.01984	3.23	0.3146	415	0.02379	102.75

*s = (π/8) (K_Ic_/σ_F0_)^2^; **σ_FC_ = σ_F0_ √([s/c]/{1 + [s/c]}); ^σ_FC_ =(√8/π)σ_F0_√(s/c)

**Figure 17:**
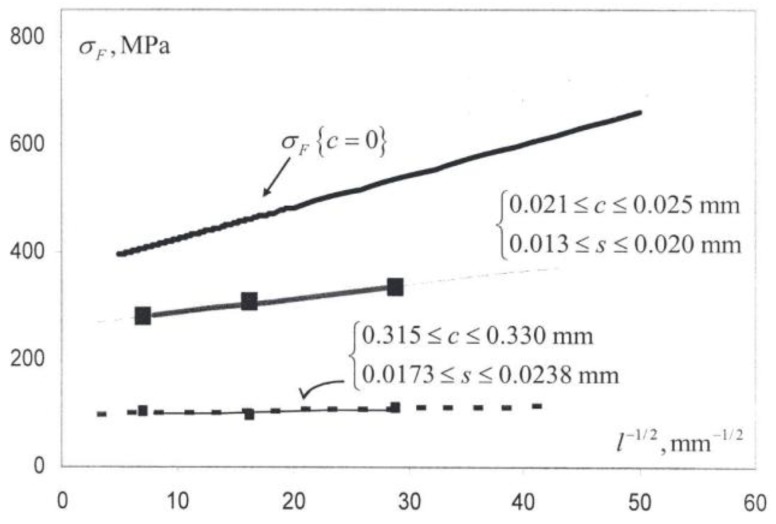
Crack-free and pre-crack H-P fracture stresses for Al_2_O_3_ [1].

**Figure 18:**
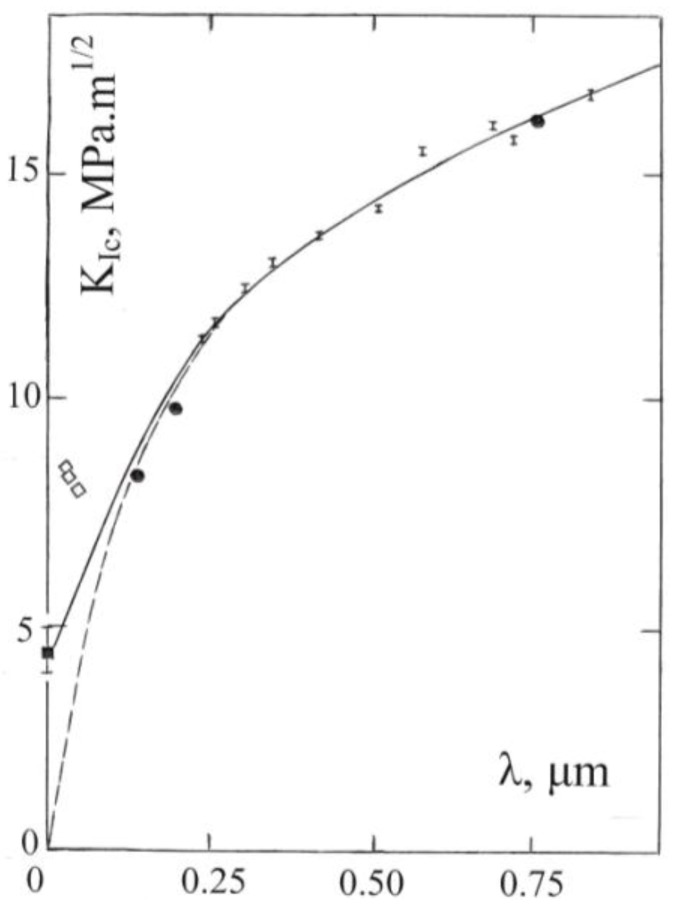
A compilation of conventional and indentation fracture mechanics K_Ic_ measurements reported for WC-Co material [42,54,55,56] based on the Co mean free path, λ, dependence given in [54].

Presentation of comparable fracture toughness properties of WC-Co material begins with the reminder of toughness decreasing at smaller WC particle sizes for those materials shown in Figure 15 [42]. In addition, toughness decreases with reduction of the mean free path, λ, of the binder Co phase. Figure 18 shows an updated compilation of results [42,54,55,56].

**Figure 19:**
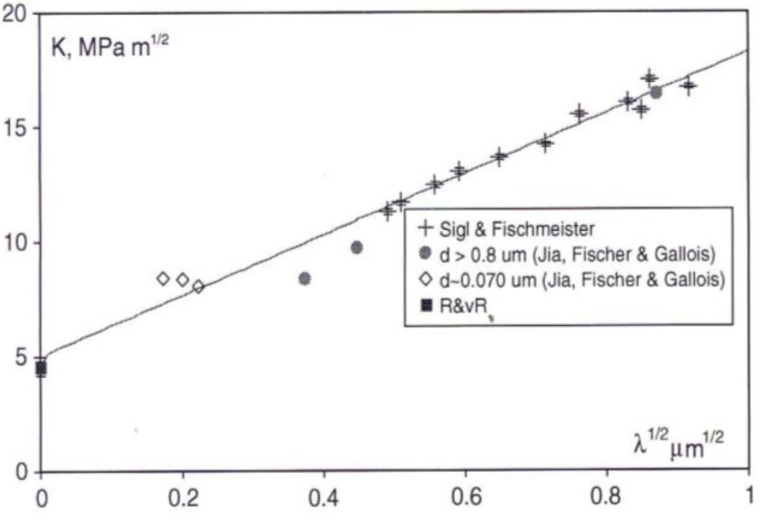
Conventional and indentation fracture mechanics measurements for WC-Co [1,42,55,56] based on Equation (1) with λ taken proportional both to the plastic zone size, s, and the particle size, ℓ.

The abscissa and ordinate scales of Figure 18 have been extended from the previously reported figure [54] including only the important points shown as vertical short-line-segments [55]. These data were obtained in bend tests and the K_Ic_ measurements were computed on the basis of constructing a detailed account of the energies dissipated during the process of fracturing. Contiguity of the WC particles was taken into account. The fracturing energy was found to be governed by plastic dissipation associated with a “multiligament” path length spreading either within the Co binder or near to the binder/particle interfaces. The H-P dependence described for the Co phase from Equation (6) and in Figure 13 was taken into account in assessing the plastic work. In the original model description for which Figure 18 was constructed, the fracturing mechanism was described in terms of that two-part multiligament zone as compared with the earlier-mentioned plastic zone size, s, in Equation (1). Nevertheless as shown in the graph for the total measurements now also contained in Figure 19, the K_Ic_ measurements were able to be plotted in the form of Equation (1) with s and the grain size, ℓ, taken to be proportional to the cobalt mean free path, λ, so that

K_Ic_ = α_1_[σ_0C_λ^1/2^ + α_2_k_C_] (7)

The linear λ^1/2^ dependence shown in Figure 19 for K_Ic_ has been added to by incorporating other WC-Co results obtained at conventional (filled circle) and nanometer (open diamond) grain sizes for WC-Co materials [56] and for a toughness result reported for WC material [42], as also shown in each case in Figure 18. The added K (=K_Ic_) measurements were obtained in IFM tests. The fracture paths of the nanostructured materials were shown also to follow a network of bridged ligaments consistent with the earlier results obtained at larger WC particle sizes [55]. Other K measurements involving agreement between microindentation and three point bending test measurements have been reported for Al_2_O_3_ and related ceramic materials [57].

Elevated temperature measurements over 20 to 1000 C have been reported for bend tests performed on WC-Co materials with micron and sub-micron WC particle sizes [58]. Measurements of the temperature dependence of the transverse rupture stresses for the micron particle size material followed the general trend indicated here in Figure 6 and Figure 11 also with fall-off in strength in the range of 800–900 K. A ductile-brittle transition (d-bt) just below those temperatures was associated with intersection of the rupture strength and yield stress curves. Significant plastic straining prior to fracturing occurred above the d-bt temperatures. Complete stereological measurements were reported. The ambient temperature hardness and fracture stress measurements in [58] were compared in [1] with estimations following on from Equation (4) and Figure 17, allowing for a proportional relationship between ℓ and λ for the tested material compositions. Again, fracturing was attributed largely to failure of the Co binder phase. An interesting report [59] relating to influence of temperature, while in agreement with the importance of the strength properties of the Co binder phase, has attributed a special role to thermal stresses associated with different thermal expansions of the phases in determining the material fracture toughness. The reported model for influence of the thermal expansion incompatibility on fracturing energy was shown to be consistent with an H-P dependence of the fracture stress and to give a similar dependence of the fracture mechanics stress intensity K value to that expressed in Equation (7) involving the Co binder mean free path. Such thermal stress consideration in the WC-Co system is given weight in a report of calculated micro-stresses and their measurement by x-ray and neutron diffraction methods [60].

## 7. Summary

The hardness and strength properties of WC-Co materials have been reviewed with the aid of related results obtained on Al_2_O_3_ and MgO crystals and polycrystals. First, microindentation and nanoindentation hardness test measurements were compared on a hardness type stress–strain basis that included description of the elastic, plastic and cracking behaviors of crystals. Then, consideration was given to a role for the importance of temperature in establishing the plastic strength properties of MgO, Al_2_O_3_ and WC crystals and WC-Co materials. A contiguity, C, modified Hall-Petch (H-P) relationship, taking into account WC-to-WC particle contacts, was shown to describe satisfactorily an increased hardness of WC-Co with decreased particle size while also thereby giving greater weight to the hardness properties of the Co binder phase. And, with important scatter of results to be taken into account, a relationship was established between the fracture strengths and fracture mechanics toughness properties of Al_2_O_3_ and WC-Co materials, in both cases, also with influence of crack sizes, grain sizes, and plastic zone sizes. All of the results obtained on the WC-Co composite system point to the importance of the Co binder mean free path dimensions in effecting the composite material toughness properties, finally mentioning their role in the fracturing behavior of internal thermal micro-stresses.
